# Impact of N6-methyladenosine (m^6^A) modification on immunity

**DOI:** 10.1186/s12964-022-00939-8

**Published:** 2022-09-09

**Authors:** Raghda A. Elsabbagh, Mona Rady, Carsten Watzl, Khaled Abou-Aisha, Mohamed Z. Gad

**Affiliations:** 1 Biochemistry Department, Faculty of Pharmacy and Biotechnology, The German University in Cairo, Cairo, Egypt; 2 Microbiology, Immunology and Biotechnology Department, Faculty of Pharmacy and Biotechnology, The German University in Cairo, Cairo, Egypt; 3 Faculty of Biotechnology, German International University, New Administrative Capital, Cairo, Egypt; 4 Department of Immunology, Leibniz Research Centre for Working Environment and Human Factors (IfADo)-TU Dortmund, Dortmund, Germany

**Keywords:** N6-methyl-adenosine, Innate, Adaptive, Immunity, m^6^A writers, m^6^A erasers, m^6^A readers

## Abstract

N6-methyl-adenosine (m^6^A) is the most prevalent modification on mRNAs and long noncoding RNAs (lnRNAs) in higher eukaryotes. Modulation of m^6^A relies on m^6^A writers, erasers and readers. m^6^A modification contributes to diverse fundamental biological functions at the molecular, cellular, and physiological levels. The dysregulation of m^6^A modification has been implicated in various human diseases. Thus, m^6^A modification has now become a research hotspot for its potential therapeutic applications in the treatment of various cancers and diseases. The immune system is essential to provide defense against infections and cancers. This review summarizes the current knowledge about the roles of m^6^A in regulating immune cell functions and immune responses.

**Video abstract**

**Supplementary Information:**

The online version contains supplementary material available at 10.1186/s12964-022-00939-8.

## Background

The first modification in DNA nucleotides was discovered in 1948 [1] and since then the “epigenetics” research field has developed and evolved. Over time, the contributions of epigenetics in almost all cellular functions through regulation of gene expression became evident. Our knowledge has extended to post-translational protein modifications which are now well recognized to control the proteins’ fate. In contrast to DNA and proteins, RNA was considered to be less important and thought to merely be a transitional element bridging the information stored in the DNA and the synthesized proteins [2].

**Fig. 1:**
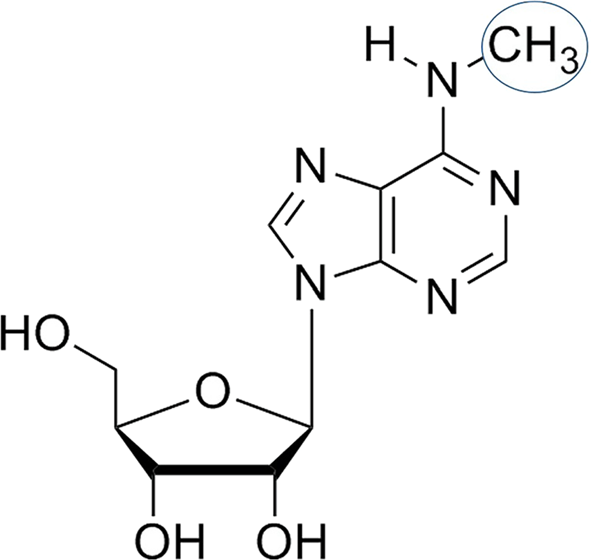
N6-methyladenosine (m6A) modification

It was later on discovered that 70–90% of the human genome is transcribed into RNA but only 1–3% of the transcriptome actually bears the blueprint for the synthesis of proteins [2]. It was not until the 1980s when light was shed upon the functions of RNA molecules, other than coding for a peptide. Since the emergence of next-generation sequencing (NGS) technology, research was shifted extensively towards the epitranscriptome which represents the biochemical base modifications of a cell’s RNA transcripts that are not genetically encoded in the RNA sequence [2, 3]. So far, more than 100 RNA modifications have been identified in different types of RNA [4]. Those modifications modulate nearly all aspects of RNA metabolism and the associated physiological processes making them a key component of the post-transcriptional gene regulatory landscape [2, 3]. Among these RNA modifications, the N6-methyladenosine (m^6^A) (Fig. 1) represents the most prevailing post-transcriptional modification in eukaryotic RNA transcripts as well as long noncoding RNAs (lncRNAs) [5].

Serendipitously discovered in 1974 [6, 7], the m^6^A modification refers to the post-transcriptional methylation of the mRNA adenine base at the nitrogen-6 position [8]. After the emergence of antibody-based immunoprecipitation followed by high-throughput m^6^A sequencing (MeRIP–Seq), it was revealed that human mRNA transcripts are punctuated by m^6^A at highly conserved and specific sites, particularly in the vicinity of stop codons, at the 3′ untranslated regions (3' UTRs), and in consensus sequences within long exons [9]. There are two slightly differing consensus motifs proposed in which m^6^A occurs: DR m^6^A CH [10] and RR m^6^A CH [11, 12] (with D = G, A, or U; R = G or A; and H = C, A, or U).

m^6^A modification modulates RNA secondary structure/folding. These m^6^A—derived alterations of target RNA structures are directly conveyed to their fates, functions and metabolism. These include (a) altering the mRNA splicing pattern, thereby potentially changing the distribution of splice isoforms of the transcript depending on the tissue or organ, (b) modulating the intracellular distribution and localization of mRNA by affecting nuclear export/retention, (c) influencing the potential for translation; or (d) impacting the stability of the transcript affecting its decay rate [8]. Moreover, m^6^A modification on chromosome-associated regulatory RNAs (carRNAs), which include promoter-associated RNAs (paRNA), enhancer RNAs (eRNA), and repeat RNAs, regulate chromatin accessibility and downstream transcription [13]. m^6^A could make the chromatin more or less accessible, thus increase or decrease transcription and translation [14–17]. m^6^A methylation also modulates the status of histone methylation or acetylation, and subsequently, histone modification can tune gene expression [13]. Any of these processes consequently leaves a print on the potential of translation, thereby affecting both the nature and quantity of the various produced protein isoforms. These molecular effects are then conveyed to the cellular level by influencing cell metabolism, circadian rhythm [18], cell differentiation [19–22], reprogramming, state transitions and stress responses [20, 23, 24], thus shaping cell function and identity. These effects are sequentially echoed to the organism’s physiology [3, 25]. Thus, a disturbance in the balance of m^6^A modifications can result in abnormalities in transcripts and proteins levels which are associated with various diseases and types of cancers [2, 26–30].

The immune system is the human body’s defense weapon against microbes and cancers. Through immunological surveillance, the immune system uses different mechanisms to recognize and combat the broad range of pathogens it encounters. This monitoring process of the immune system is also extended to the detection of virally infected, stressed, transformed and malignant cells making the immune system a key player in fighting infections and cancers [4].

m^6^A modification adds another layer of regulation to the already sophisticated gene expression regulation pathways in mammals. Extensive research has been carried out on the regulatory roles of m^6^A in stem cells and cancer cells. However, only little is known about the role of m^6^A in the immune system. In this review, we summarize the recent findings on the impact of m^6^A in different types of immune cells.

## Protein factors involved in cellular m^6^A methylation

Adenosine methylation is a dynamic and reversible process that is orchestrated by extremely conserved methyltransferases (“writers”) and demethylases (“erasers”). Together, the writers and erasers shape the cellular ‘epitranscriptome’. The methyl code is decrypted by a cluster of m^6^A readers which sequentially direct the fate of the modified transcripts. The dynamic interplay between the writers, erasers and readers create the methylated transcriptome and dictate the prevalence, distribution and the m^6^A-dependent functions on RNA [3, 8]. Recently, with the development of advanced m^6^A detection methods [31, 32], scientists began to unveil the full repertoire of m^6^A proteins and how they finely contribute to the tuning of mRNA and lncRNA regulation.

## m^6^A writers—adenosine methyltransferases

The m^6^A modification is catalyzed by the m^6^A writer complex inside the nucleus, which consists of the enzymatically active methyltransferase-like 3 (METTL3) protein and some interacting proteins. METTL3 was the first identified of all core writer components, first reported in 1994 as an *S*-Adenosyl methionine-binding protein with methyltransferase capacity. Known interaction partners of METTL3 are: METTL14, Wilms' tumor 1-associating protein (WTAP), KIAA1429 and RNA-binding motif protein 15 (RBM15). METTL3 activity was also detected in the cytoplasm where it acted to promote translation independent of its methyl transferase activity [9]. METTL14 doesn’t catalyze methyl-group transfer. Rather, it forms a stable heterodimer with METTL3 in a stoichiometric 1:1 ratio and acts as the RNA binding platform which binds to substrate mRNA to enhance the enzymatic activity of METTL3. Separately, METTL3 and METTL14 show comparable weak methyltransferase activity in vitro; synergistically, they exhibit a much higher catalytic activity [2, 27, 33].

WTAP is an essential component of the writer complex. As it lacks methyltransferase domains, it acts as an adaptor protein translocating the METTL3-METTL14 complex to mRNA. Likewise, RBM15 and RBM15B interact with METTL3 in a WTAP-dependent way using their RNA-binding domains enabling the writer complex to bind to specific mRNAs. KIAA1429 is another accessory component associated with the writer complex. KIAA1429, also known as, protein virilizer homolog VIRMA, was reported to guide the METTL3-METTL14 heterodimer to mRNA for region-selective m^6^A methylation. METTL16 was recently described as a methyltransferase exerting its functions independently of the m^6^A writer complex surrounding METTL3 [2, 27, 33].

## m^6^A erasers—demethylases

Fat mass and obesity-associated protein (FTO) and alkylated DNA repair protein alkB homologue 5 (ALKBH5) are the two m^6^A demethylases identified to date [25, 34]. Both proteins are members of the AlkB family, each displaying distinct subcellular and tissue distributions. FTO is readily detected in both the nucleus and the cytosol [6]; however, ALKBH5 is markedly enriched in the nucleus. Hence, this implies that FTO is capable of targeting mature RNAs regulating cytosolic mRNA processing events and ALKBH5 may target nuclear mRNAs where it can regulate export and metabolism of mRNA [27, 33]. It was reported that inactivating ALKBH5 increased total m^6^A mRNA levels and this was accompanied by accelerated nuclear export and accumulation of mRNA in the cytoplasm [35]. Consistent with these findings, a lack in m^6^A slowed down nuclear export, delaying the nuclear exit and elongating nuclear retention times [18]. The enzymes’ tissue distribution is another difference between the two enzymes. FTO is broadly expressed in all adult and fetal tissues and highly enriched in brain tissue [36], whereas ALKBH5 is mostly expressed in testes and at substantially lower levels in other tissues [35]. This might suggest that ALKBH5 imparts its demethylase activity in tissues that lack FTO and vice versa [6]. FTO was the first m^6^A eraser identified several decades ago [36]. FTO was first reported to associate with increased body mass and obesity in humans [37, 38]. Overexpression of FTO in mice led to decreased total m^6^A levels accompanied with increased food intake, body weight and fat mass [25].

## m^6^A readers— m^6^A RNA binding proteins

The m^6^A modifications are recognized by m^6^A readers and investigating them has shed light on the role of m^6^A in RNA processing [27]. The m^6^A modifications are predominantly read either by proteins that are members of the YT521-B homology domain-containing family (YTHDF) and interact with m^6^A sites via their YTH domains or by the eukaryotic initiation factor 3 (eIF3) [31]. YTH-containing reader proteins include YTH N6-methyladenosine RNA binding proteins 1/2/3 (YTHDF1, YTHDF2, YTHDF3) and YTH Domain-Containing Protein 1 (YTHDC1) which have a conserved m^6^A-binding pocket [3]. YTHDF1 recruits m^6^A‐containing transcripts to ribosomes by interacting with translation initiation factors, thereby promoting translation [31]. However, the m^6^A sites on a transcript seem to be decisive on its fate; whereas methylation within transcripts’ UTRs promoted translation, methylation within coding regions attenuates translation [39].

In contrast, YTHDF2 speeds up the degradation of 3' UTR m^6^A-modified mRNA transcripts by either interfering with the binding of mRNA stabilizing proteins or by recruiting proteins, which target mRNAs to processing bodies (the cellular sites of mRNA decay) [6, 40].

It was revealed that both the YTHDF1 and YTHDF2 m^6^A readers share a similar set of target transcripts and cooperate harmoniously. Interestingly, YTHDF1 binds to mRNA in the early stage of its life to promote translation as long as the protein is required. YTHDF2 then associates with the transcripts after their cellular duties are accomplished to alter their stability and sentence them to decay [3, 25]. A study demonstrated that m^6^A methylation appeared to promote the protein expression of one transcript and to downregulate another. They then explained that the former transcript was a target of YTHDF1 and the latter was a target for YTHDF2 [41].

On the other hand, m^6^A might also stabilize mRNAs by binding to certain reader proteins that encourage transcript stability preventing their degradation and naturally increasing their expression [42, 43]. Recently, a study stated that insulin-like growth factor 2 mRNA-binding proteins (IGF2BPs, including IGF2BP1/2/3) could also recognize m^6^A modifications, and can be considered as a distinct family of m^6^A readers. IGF2BPs exhibits mRNA – stability promoting functions in an m^6^A-dependent manner in contrast to the mRNA-decay-promoting function of YTHDF2 [44].

**Fig. 2:**
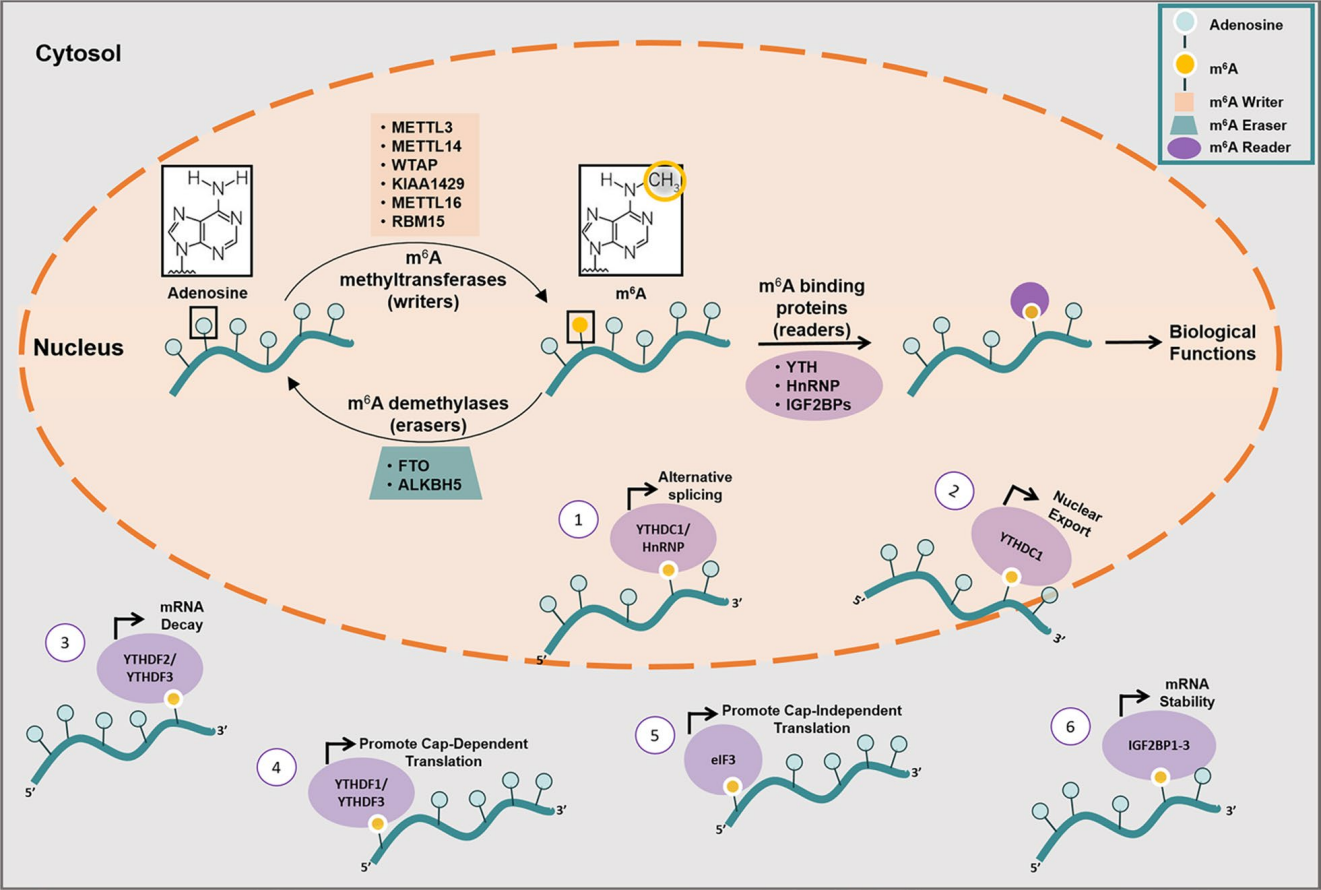
The dynamics of the m6A methylome. The methylated adenosine group is colored yellow. m6A writers, erasers and readers are the protein factors involved in the cellular m6A methylation event. m6A writers catalyze the covalent conversion of Adenosine (A) to m6A on target RNAs. FTO and ALKBH5 are m6A erasers that reverse the methylation. A diverse set of m6A readers selectively bind m6A and mediate post-transcriptional processes on m6A-containing RNA including ① alternative splicing ② nuclear export and RNA localization ③ mRNA degradation ④ 7-Methylguanosine cap-dependent translation ⑤ 7-Methylguanosine cap-independent translation ⑥ and mRNA stabilization

YTHDF3, cooperating with YTHDF1 and YTHDF2, can regulate mRNA translation and mediate mRNA decay, respectively [27]. Last but not least, YTHDC1 is the main reader of nuclear m^6^A modifications. It’s present in both nucleus and cytoplasm and is characterized as a modulator of mRNA splicing events. Additionally, the heterogeneous nuclear ribonucleoprotein (hnRNP), another m^6^A reader, binds to m^6^A-containing pre-mRNAs and has been shown to affect alternative splicing [2]. eIF3 is a central player in the recruitment of the pre-initiation complex (PIC) to mRNA and the initiation of translation [45].  \* MERGEFORMAT Fig. 2 summarizes the key players involved in the cellular m^6^A methylation events.

## m^6^A and the innate immune response

The m^6^A methylation events appear to be integral in the functioning of the innate immune response. Studies demonstrate that m^6^A modification tightly controls various innate immune responses such as the expression of interferons (IFNs), inflammatory responses, and macrophages and dendritic cells homeostasis. m^6^A can either improve the immune response against pathogens and viruses or tame the immune response to prevent aggressive immunopathological damage [46].

### ***m***^***6***^***A in NK cells***

NK cells are innate immune lymphocytes with natural cytotoxicity and cytokine-producing effector functions. It was discovered that m^6^A methylation acts a positive regulator of NK cell antitumor and antiviral activities. Upon activation by cytokines, tumors, and virus infection, YTHDF2 is upregulated in NK cells. YTHDF2 maintains NK cell homeostasis, maturation and IL-15–mediated NK survival. IL-15 is a crucial regulator of NK cell development, survival and effector functions by forming a STAT5–YTHDF2 positive feedback loop. YTHDF2 deficiency in NK cells impairs NK cell antitumor and antiviral activity in vivo. Tardbp (TAR DNA-binding protein 43 [TDP-43]) transcript is m^6^A methylated serving as an YTHDF2 binding target. Tardbp is involved in NK cell proliferation and survival. YTHDF2 regulates NK cell proliferation through modulating the mRNA stability of Tardbp and consequently its expression [47]. It was also revealed that METTL3 expression is positively correlated with levels of effector molecules in NK cells and NK effector functions. The mRNA encoding SHP-2 is m^6^A modified. METTL3-mediated m^6^A methylation of SHP-2 promotes its expression, thus mediating the activation of AKT-mTOR and MAPK-ERK signaling pathways in response to IL-15 stimulation. METTL3 depletion in NK cells impairs NK homeostasis, renders NK cells hypo-responsive to IL-15 and hinders NK cell infiltration and function in the tumor microenvironment (TME). Mice conditionally deficient for METTL3 in NK cells exhibited aggressive tumor progression, suppressed effector functions of NK cells and shortened survival time. These findings show that METTL3-mediated m^6^A methylation safeguards the homeostasis and tumor immunosurveillance of NK cells [48]. Investigating the biological roles of m^6^A modifications in NK cells will open a path to exploit NK power in antitumor immunity.

### ***m***^***6***^***A in Dendritic cells***

Dendritic cells (DCs) are antigen-presenting cells that initiate an immune response by activating T cells, thus bridging between the innate and adaptive immune systems. A study reported that m^6^A installed by METTL3 is crucial for DCs maturation and activation, thus m^6^A serves as a positive regulator. CD40, CD80, and the toll-like receptor (TLR) signaling adaptor protein (TIRAP) are crucial molecules in DCs for inducing T cell activation. METTL3-mediated m^6^A of these transcripts enhanced their translation in DCs via YTHDF1, thus promoting DCs activation and stimulating DC-mediated *T* cell activation [49]. Additionally, CCR7 chemokine receptor stimulation induces DCs migration toward draining lymph nodes. This is important for the initiation of protective immunity and maintenance of immune homeostasis. A long non-coding RNA, lnc-Dpf3, hinders the CCR7-induced DC migration and inflammatory response via inhibiting HIF1α-dependent glycolysis in DCs, therefore inhibiting their migratory capacity. m^6^A-modified lnc-Dpf3 could be degraded when it’s recognized by YTHDF2. CCR7 stimulation upregulates lncDpf3 transcripts by removing m^6^A, preventing RNA degradation. This negative feedback inhibition via lnc-Dpf3 is vital to prevent exaggerated CCR7-mediated DC migration, therefore prevents amplified adaptive immune responses and inflammatory injuries, maintaining immune balance [50].

On the other hand, recent reports show that m^6^A plays a negative role in the antitumor immune response specifically in the cross-presentation of tumor antigens for priming T cells by DCs [51]. Antitumor immunity is spontaneously generated by tumor neoantigens, but still, despite expression of neoantigens, tumors can still evade immune recognition. YTHDF1 readers in dendritic cells (DCs) recognize m^6^A -modified mRNAs encoding lysosomal proteases. They subsequently facilitate the translation of lysosomal proteases, enzymes that destroy proteins in phagosomes, thus destroy antigens, quashing thereby the cross-presentation of engulfed tumor neoantigens by DCs. This is considered one mechanism of immune evasion. Depletion of YTHDF1 in DCs in mouse models enhances cross-presentation of tumor antigens, promotes their cross-priming with CD8^+^ T and increases the infiltration of neoantigen-specific CD8^+^ T cells in the tumor microenvironment, thus enhancing antitumor immunity. Therefore, it was proposed that YTHDF1 could be a target for immunotherapy [51].

It is well known that nucleic acids can trigger the innate immune response via activation of endosomal toll-like receptors (TLRs), RIG-like receptors and cytosolic DNA sensors. Interestingly, it was also noticed that DCs treated with m^6^A-modified RNAs, produce significantly less cytokines and activation markers than when exposed to unmodified RNAs, suggesting that m^6^A impedes DCs activation. DCs and TLR-expressing cells can better detect and respond to unmodified RNAs as means of selectively responding to invading bacteria or necrotic tissue. However, they are not activated by mammalian total RNA which is m^6^A-abundant. It was also thought by some researchers that the presence of m^6^A in some viruses serve the virus in evading the host immune system [52]. Another study reported that the Influenza and Rous sarcoma viruses harbor m^6^A-modified-RNAs, and these are unable to elicit antiviral innate immune signaling and induce IFN expression [53]. Similar results were reported when the role of m^6^A in innate immunity induced by exogenous circular RNAs (circRNAs) was investigated. circRNAs prevail in eukaryotic cells and viral genomes. Foreign circRNAs are powerful adjuvants to induce antigen-specific T cell activation, anti-tumor immunity *in vivo* and antibody production. Mammalian cells possess innate immunity to detect foreign exogenous circRNAs. It was reported that m^6^A-modified human circRNAs suppress the innate immunization against “self” circRNAs, apparently protected by the m^6^A modification. On the other hand, unmodified circRNAs increase interferon production [54].

### ***m***^***6***^***A in Macrophages***

Macrophages, serving as the first line of host defense, are the scavenger cells of the innate immune system playing significant roles in autophagy by engulfing worn-out cells and other cellular debris. They also act as antigen presenting cells and secretory cells that produce a variety of cytokines vital to the host immune defense against infection [4]. m^6^A modification was also reported to play a role in macrophage functions.

Investigating the m^6^A regulatory enzymes during macrophage polarization revealed that METTL3 is upregulated during M1 polarization of mouse macrophages. Depending on their genetic background and environmental stimuli, macrophages can be polarized to one of two phenotypes, either M1 or M2, based on in vitro model systems [55]. M1 macrophages are tumoricidal, produce interferon γ (IFN-γ) with proinflammatory activity, and have a high capacity for antigen presentation and T cell activation. M2 macrophages are of a protumoral phenotype and produce interleukin-4 (IL-4) with anti-inflammatory and immunosuppressive function. Alterations in macrophage polarization between M1 and M2 phenotypes control various physiological and pathological processes. It must be kept in mind that strict M1 and M2 macrophages almost certainly do not exist in vivo since macrophages are exposed to a plethora of stimuli that result in different macrophage cell surface markers and different functions [56]. METTL3 methylates an important player in M1 macrophage polarization, STAT1 mRNA, thus upregulating its expression. METTL3 knockdown markedly reduced M1 and stimulated M2 macrophage polarization. This implies that METTL3 might play an important role in anti-inflammatory therapies [55]. Similarly, another study indicated that m^6^A and METTL3 expression levels were up‐regulated in lipopolysaccharide (LPS)‐stimulated human dental pulp cells (HDPCs) in dental pulp inflammation. In response to LPS, NF-*κ*B and MAPK pathways are activated in macrophages, which further induce the expression of various proinflammatory cytokines, such as TNF-*α*, IL-1*β*, and IL-6. Dental pulp inflammation is an inflammatory disease characterized by accumulation of inflammatory mediators. It can progress to pulp necrosis and periapical diseases, which are mainly due to a bacterial infection acting as a major pathogenic factor. METTL3 deletion decreases the expression of inflammatory cytokines and suppresses the activation of Nuclear Factor kappa B (NF-*κ*B) and MAPK signaling pathway in LPS-induced HDPCs. METTL3 was found to modulate the alternative splicing of MyD88, a splice variant of MyD88 that inhibits inflammatory cytokine production. m^6^A inhibition significantly increases MyD88S mRNA levels which consequently inhibits proinflammatory cytokines production. This suggests that METTL3 modulates LPS-induced inflammatory response of HDPCs by regulating alternative splicing of MyD88 in HDPCs [57].

Consistently, m^6^A modification also has a positive regulatory role in macrophage activation. Macrophage Toll-like Receptors (TLRs) play a vital role in sensing invading pathogens. TLR4, induce type I interferons and inflammatory cytokines such as TNF-α and IL-6. IL-1 receptor–associated kinase 3 (IRAK3), also known as IRAKM, is a negative regulator of TLR signaling pathways. The transcripts of IRAKM gene are highly m^6^A-modified. METTL3 deficiency led to the loss of m^6^A modification on IRAKM mRNA, leading to slowing down decay rate and therefore the suppression of TLR signaling–mediated macrophage activation. Loss of METTL3 promotes tumor growth, increased susceptibility to bacterial infection in vivo and reduced TNF-α secretion by macrophages in vitro. This concludes that METTL3 deficiency inhibits macrophage activation by inducing a negative regulator of the TLR signaling pathway. These findings implicate the m^6^A machinery as a potential cancer immunotherapy target [58]. Supporting these results, another study demonstrated that METTL3 deletion in macrophages promotes tumorigenesis and metastasis by enhancing tumor-associated macrophages (TAMs) and T regulatory (Treg) cells infiltration into the tumor microenvironment (TME). Most tumors shape the TME by recruiting TAMs and Tregs, which induce an immunosuppressive TME. METTL3 deficiency impairs the YTHDF1- mediated translation of SPRED2 mRNA, an m^6^A target gene. This enhances the activation of NF-kB and STAT3 through the ERK pathway in METTL3-depleted macrophages, leading to increased tumor growth and metastasis [59].

It can be concluded from the previous studies in macrophages that METTL3 is crucial for macrophage activation and for initiating a pro-inflammatory cascade or exerting a tumoricidal role. Depleting METTL3 in macrophages hindered macrophage activation, promoted anti-inflammatory and immunosuppressive activities and encouraged tumor growth and metastasis. In contrast, the m^6^A reader, YTHDF2, plays a negative regulatory role in LPS-mediated inflammatory responses of macrophages. YTHDF2 depletion results in the upregulated expression and stability of MAP2K4 and MAP4K4 mRNAs, upstream molecules in the LPS-induced inflammatory response, which promote the expression of proinflammatory cytokines. Thus, YTHDF2 can be another likely target for anti-inflammatory therapies [60].

## m^6^A and the adaptive immune response

There are two types of lymphocytes critical for the adaptive immune response, T-lymphocytes (*T* cells) and B-lymphocytes (*B* cells). They originate from stem cells in the bone marrow and differentiate in the central lymphoid organs. *T* cells mediate the cellular immune response and B cells produce antibodies in humoral immune responses [4].

### ***m***^***6***^***A modification in T cells***

D2-like Dopamine (DA) receptors, which are highly influenced by m^6^A modification events, are not only expressed in the brain but also in *T* cells. They contribute to the regulation of T-lymphocyte function and development in the thymus thus linking m^6^A modification with normal T lymphocytes development and immune responses [61, 62].

In a study by Li et al., it was shown that m^6^A methylation on mRNA controls *T* cell homeostasis. Depletion of METTL3 in mouse *T* cells upsets *T* cell homeostasis and differentiation. *T* cells fail to undergo homeostatic expansion and remain in the naive state for up to 12 weeks. m^6^A mRNA methylation targets the IL-7/STAT5/SOCS pathways, which represent an important signal axis in the maintenance of *T* cell proliferation and differentiation. Deleting METTL3 decreased methylation of the Suppressors of Cytokine Signaling (SOCS) family genes transcripts, which encode the IL-7/STAT signaling inhibitory proteins. These hindered the mRNA decay and increased mRNAs, mRNA half-life and SOCS protein expression in naive *T* cells. The amplified activity of the SOCS family consequently inhibited IL-7-mediated STAT5 activation and suppressed *T* cell homeostatic proliferation and differentiation. This means that m^6^A is essential for inducing decay of SOCS mRNAs, in order for *T* cells to escape the naïve state in response to IL-7/STAT signaling [63]. Building up on the previous study, researchers quantified RNA dynamics in *T* cells, using bioinformatic analysis, to reveal how transcripts are regulated by m^6^A. In the context of *T* cell homeostasis, m^6^A depletion is reported to globally slow down the rates of all stages of the RNA life cycle by delaying RNA synthesis rates, impairing RNA processing rates and hindering SOCS mRNA decay rates. All these effects may directly or indirectly upset *T* cell differentiation [64]. Interestingly, these findings suggest that *T* cell-targeted delivery of m^6^A modifying agents could be an eminent step in cancer immunotherapy [31, 63, 65].

Likewise, research on regulatory *T* cells showed how m^6^A plays a role in their function. Regulatory *T* cells are a subpopulation of CD4^+^ *T* cells that act to reduce inflammation, suppress the immune response and reduce autoimmunity [66]. m^6^A is critical to sustain the suppressive functions of Tregs. Decreased m^6^A portrayed a similar scenario as observed in CD4^+^ naïve *T* cells. Low m^6^A led to a loss in Tregs suppressive functions where SOCS activity increased, inhibiting the IL2-STAT5 pathway, which is critical for the Treg cell functions. When Tregs with depleted m^6^A were co-cultured with naïve CD4^+^ *T* cells, it was revealed that naïve *T* cells exerted faster proliferation due to complete lack of suppressive action of Tregs. Moreover, METTL3-knockout mice develop severe systemic autoimmune diseases. It was suggested that since Tregs alleviate the tumor-killing functions of CD8^+^ *T* cells in the tumor microenvironment, the selective reduction of m^6^A in tumor-infiltrated Tregs may be advantageous in combination with other methods of cancer immunotherapy [67].

Follicular helper T (Tfh) are a unique CD4^+^ *T* cell subset and have an eminent role in the formation of germinal centers (GCs) and mediating humoral immunity. Inducible costimulator (*icos*) is crucial for Tfh development. GAPDH, a glycolytic enzyme, is a key player in regulating Tfh cell development, acting as an epigenetic regulator. GAPDH alters the METTL3/METTL14-mediated m^6^A modification on *icos* mRNA during the initiation of Tfh cells. It negatively controls *icos* gene expression, by promoting *icos* mRNA degradation via the m^6^A modification on *icos* mRNA, thus suppressing Tfh development [68].

### ***m***^***6***^***A modification in B cells***

The role of m^6^A in B cells is still under-explored. It was reported that m^6^A methylation is vital in early *B* cells development as it induces IL-7 mediated pro-*B* cell proliferation as well as the transitioning from large-pre *B* cells to small- pre-*B* cells. Deletion of METTL14 severely impairs both processes and causes defects in gene expression important for B cell development [69].

**Fig. 3:**
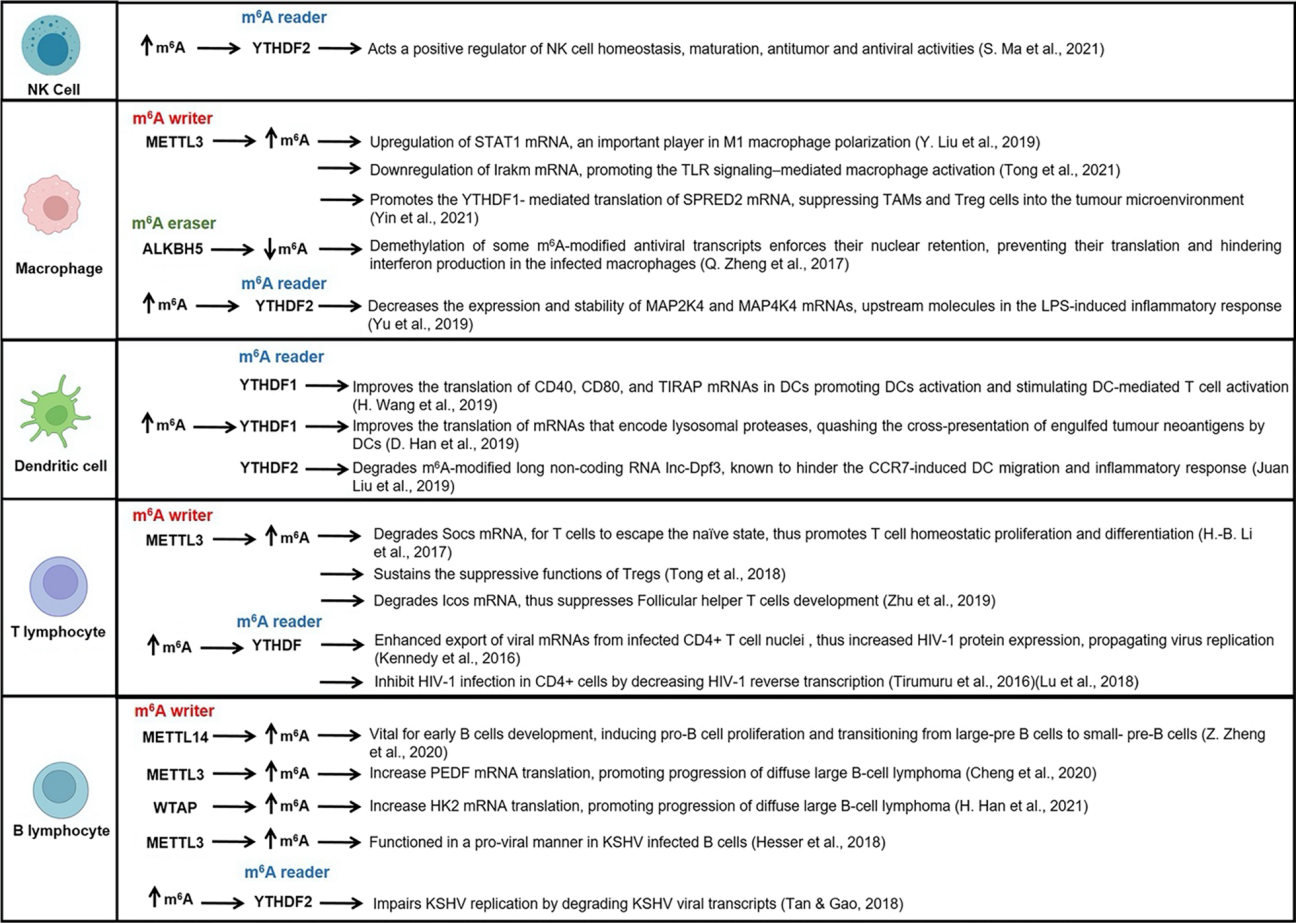
m6A regulatory pathways in some immune cells that have been revealed

The effects of m^6^A modifications on *T* cells and *B* cells have extensive implications in the adaptive immune response and surely have an impact in the development and progression of various immune-related diseases. \* MERGEFORMAT Fig. 3 shows a summary of some m^6^A regulatory pathways in some immune cells.

### ***m***^***6***^***A and antiviral immunity***

Recent research has demonstrated that the m^6^A machinery is involved in the host response to viral infection, playing either a pro-viral or an anti-viral role.

m^6^A modification can suppress the antiviral innate immune system by targeting type I interferons. The mRNA of IFN-*β*, the main type I interferon in non-immune cells that drives the type I interferon response, is m^6^A modified. m^6^A decorating the IFN-*β* dictates the fast turnover of interferon mRNAs consequently facilitating viral propagation. IFN-*β* transcripts were stabilized following repression of METTL3 or YTHDF2. Deletion of METTL3 or YTHDF2 reader led to an increase in the induction of interferon-stimulated genes following a viral infection or after stimulation with an inactivated virus. Consequently, propagation of different viruses was suppressed in an interferon-signaling-dependent manner [70]. Moreover, YTHDF3 also suppresses IFN response by upregulating FOXO3 translation. FOXO3 acts as the IFN transcription repressor [71]. These findings suggest that m^6^A serves as a negative regulator of interferon response, thus the antiviral response. Another finding shows that m^6^A acts as a negative regulator for the Rig-like receptors (RLR)-mediated sensing pathway of the Vesicular Stomatitis Virus (VSV) dsRNA. Upon VSV infection, METTL3 increases m^6^A on virus-derived transcripts and decreases viral dsRNA formation, thus reducing the virus-sensing efficacy of RLRs and attenuating the antiviral immune signaling. METTL3 depletion in the monocytes of a murine model protects the mice against VSV infection, enhances type I IFN expression and speeds up VSV clearance [72].

On the contrary, other studies reported that m^6^A is vital for the antiviral innate immune response. m^6^A modification plays a critical role in increasing the IFN release in macrophage- mediated antiviral immunity. DEAD-box (DDX) helicase members have been verified to sense viral RNAs, thus are crucial for the initiation of antiviral innate immunity. However, one of the nuclear DDX family members, DDX46 was shown to negatively regulate the production of type I interferon after viral infection. It does this by recruiting ALKBH5 to demethylate some m^6^A-modified antiviral transcripts. This enforces the nuclear retention of antiviral transcripts, preventing their translation and hindering interferon production in the infected macrophages. This might help to prevent the over activation of antiviral innate responses. In vivo knockdown of DDX46 enhances macrophage-mediated antiviral response [73]. Coincidentally, another study revealed that m^6^A modification augments the IFN- mediated antiviral immune response by encouraging the translation of certain IFN-stimulated genes (ISGs) [74]. Upon viral infection, m^6^A writer, WTAP, is degraded via the ubiquitination- proteasome pathway. This reduces m^6^A levels on the IFN‐regulatory factor 3 (IRF3) and interferon alpha/beta receptor subunit 1 (IFNAR1) mRNAs, which are transcripts crucial for IFN-derived antiviral response. Consequently, their translation is suppressed, thereby blocking IFN‐I‐mediated antiviral responses. Thus, m^6^A induced by WTAP is essential to maintain the protein abundance of IRF3 and IFNAR1, thus sustaining the antiviral response [75].

m^6^A levels also were found to be upregulated in primary human foreskin fibroblasts upon infection with human cytomegalovirus (HCMV), exhibiting a pro-viral role by activating viral propagation. Post infection, in METTL3-depleted cells, the decreased m^6^A leads to increased mRNA stability of Interferon- *β* (IFN-*β*) and sustained IFN-β production. This prompts an intense antiviral response to block HCMV growth [70]. Consistently, METTL14 depletion enhanced IFN expression and reduced viral propagation, but ALKBH5 depletion had an opposite effect [76]. Moreover, a proviral role of m^6^A machinery has also been observed with influenza A virus. Though the mechanism is unknown, it is assumed that YTHDF2 promotes the degradation of antiviral transcripts [77]. Additionally, m^6^A modification of the SARS-CoV-2 genome was investigated in regulating the innate immune response. Depleting METTL3 decreases m^6^A in SARS-CoV-2 and host genes, and this subsequently enhances the downstream innate immune signaling and inflammatory gene expression towards the virus. This shows that m^6^A has a pro-viral role suppressing the innate immune signaling [78]. Similarly, another study also reported that RBM15, a methyltransferase, was significantly elevated in SARS-CoV-2 infected patients, as well as positively correlated with disease severity. RBM15 elevated m^6^A modifications of multi-target genes thus negatively regulated host immune response to SARS-CoV-2. These findings indicate that RBM15 can serve as a target for the treatment COVID-19 [79]. HIV-1 infection of the human CD4 + T cells triggers a massive increase in m^6^A in both host and viral mRNAs. m^6^A on the viral transcripts positively correlate with HIV-1 viral replication, where m^6^A is vital for the export of viral mRNAs from T cell nuclei and subsequently viral replication. Silencing m^6^A writers decreases HIV-1 replication and silencing m^6^A erasers increased HIV-1 replication [80]. Consistently, YTHDF overexpression enhanced HIV-1 protein and RNA expression, propagating virus replication in CD4 + T cells. YTHDF downregulation reversed this effect. These results suggest that m^6^A writers and readers have pro-viral roles [81]. Conversely, another study showed that YTHDF readers recognize m^6^A-modified HIV-1 RNA and inhibit HIV-1 infection in CD4 + cells by decreasing HIV-1 reverse transcription. Knocking down YTHDF proteins had opposite effects. This implies that YTHDF can also act as a negative regulator of the HIV-1 replication, indicating that the m^6^A-mediated functions in regulating HIV-1 infection depend on different stages of the viral life cycle [82, 83].

The function of m^6^A modifications in the oncogenic human DNA virus Kaposi's sarcoma-associated herpesvirus (KSHV) remains controversial. m^6^A levels were reported to be significantly increased in B cells infected with KSHV. METTL3 and YTHDF2 functioned in a pro-viral manner and depleting them significantly reduced virion production in KSHV infected B cells [84]. Additionally, YTHDC1 encourages KSHV lytic replication by facilitating the splicing of the replication transcription activator (RTA) [85]. On the other hand, a study reported that YTHDF2 impairs KSHV replication by degrading KSHV viral transcripts [86]. Interestingly, m^6^A can be a novel target to develop new KSHV antiviral therapies.

To sum up, it is clear that m^6^A is neither consistently pro-viral nor anti-viral. Instead, it regulates many aspects of viral replication and the immune response signaling pathways by modulating specific RNAs according to the cell type [87].

## Conclusion

Recently, m^6^A modification is becoming one of the hot spots of life sciences gaining vast attention of RNA biologists because of its various functional implications [29]. The dynamic interplay between the methyl writers, readers and erasers creates an optimally methylated transcriptome that dictate the m^6^A-dependent functions and fate of RNA [3]. m^6^A can modulate the mRNA life cycle transcriptionally and post-transcriptionally, which include pre-mRNA processing, export, translation and decay processes.

It was noted that m^6^A could affect diseases by regulating the immune system, unfolding the curtains on the link between m^6^A and immunotherapy. Targeting the m^6^A modification could enhance the patient’s own immune system to fight against progressive cancers and other diseases. Thus, m^6^A could be potential pharmacological targets [33, 88].

In this review, we summarized some recent findings of m^6^A modification in immune cells. In general, we concluded that the role of m^6^A in various immune cells is controversial. Interestingly, m^6^A can potentially exert dual opposite effects on the fate of methylated transcripts. The fate of m^6^A modified transcripts is dictated by several factors. One factor is governed by which type of reader protein recognizes and binds to the transcript at which time point. Different readers may target different set of transcripts but sometimes, different readers may preferentially bind to diverse regions within the same transcripts or may even compete on the same region within the same transcript. Therefore, to better understand the m^6^A mediated regulation of mRNA transcripts, it is important to know which regions of the transcripts are m^6^A modified and which readers bind to the modified sections [89]. Another factor is that m^6^A regulatory proteins may function differently in different cell context by regulating different sets of targets, concluding that m^6^A regulation is of cell heterogeneity [90]. Thus, it is not unusual that an m^6^A writer and an eraser may exert the same result in a given type of pathological condition, probably through targeting distinct sets of genes [29]. Alternatively, they may also regulate the same set of target genes and cause similar biological effects via different mechanisms [89], so each case must be analyzed individually. The greatest challenge is that m^6^A is a dynamic and reversible modification, so pinpointing the exact modification sites and the key transcripts regulated by m^6^A is difficult. Moreover, any manipulation of m^6^A to manipulate the immune response will be difficult and will need to be specifically targeted.

How and when are m^6^A regulatory proteins involved in the methylation event? How do they interact with one another? Do the roles of m^6^A work in concert or are antagonistic in different immune cells? In other words, can the effect of m^6^A in the different immune cells result in a general immunosuppressant or an immunostimulant effect? How and why do m^6^A regulators mediate specific gene expression regulation? All these questions are still unresolved. We anticipate that more extensive research on m^6^A in immune cells and the immune response will open the door for exploiting immune cells in novel therapeutic strategies including cancer immunotherapy, antiviral, anti-inflammatory and autoimmune disease therapies.

## Data Availability

Not applicable.

## Abbreviations

3' UTR: 3' Untranslated Region
ALKBH5: Alkylated DNA repair protein alkB homologue 5
AML: Acute myeloid leukemia
DA: Dopamine
DC: Dendritic cell
DDX: DEAD-box
DLBCL: Diffuse large *B*-cell lymphoma
FTO: Fat mass and obesity-associated protein
GSCs: Glioblastoma stem cells
HCC: Hepatocellular carcinoma
HCMV: Human cytomegalovirus
IFN: Interferon
IGF2BPs: Insulin-like growth factor 2 mRNA-binding proteins
IL: Interleukin
IRAK3: IL-1 receptor–associated kinase 3
KIAA1429: Protein virilizer homolog VIRMA
KSHV: Kaposi's sarcoma-associated herpesvirus
LPS: Lipopolysaccharide
METTL3: Methyltransferase-like 3
NK cells: Natural Killer cells
NSCLC: Non-small-cell lung carcinoma
PEDF: Pigment epithelium-derived factor
PIC: Pre-initiation complex
piRNAs: PIWI-interacting RNAs
RTA: Replication transcription activator
SOCS: Suppressors of cytokine signaling
Tfh: Follicular helper T
TIRAP: TLR signaling adaptor protein
TLR: Toll-like receptor
TME: Tumor microenvironment
TNF: Tumor necrosis factor
WTAP: Wilms' tumor 1-associating protein
YTHDF: YT521-B homology domain-containing family
